# Meiotic Recombination Hotspots of Fission Yeast Are Directed to Loci that Express Non-Coding RNA

**DOI:** 10.1371/journal.pone.0002887

**Published:** 2008-08-06

**Authors:** Wayne P. Wahls, Eric R. Siegel, Mari K. Davidson

**Affiliations:** 1 Department of Biochemistry and Molecular Biology, University of Arkansas for Medical Sciences, Little Rock, Arkansas, United States of America; 2 Department of Biostatistics, University of Arkansas for Medical Sciences, Little Rock, Arkansas, United States of America; University of Munich and Center of Integrated Protein Science, Germany

## Abstract

**Background:**

Polyadenylated, mRNA-like transcripts with no coding potential are abundant in eukaryotes, but the functions of these long non-coding RNAs (ncRNAs) are enigmatic. In meiosis, Rec12 (Spo11) catalyzes the formation of dsDNA breaks (DSBs) that initiate homologous recombination. Most meiotic recombination is positioned at hotspots, but knowledge of the mechanisms is nebulous. In the fission yeast genome DSBs are located within 194 prominent peaks separated on average by 65-kbp intervals of DNA that are largely free of DSBs.

**Methodology/Principal Findings:**

We compared the genome-wide distribution of DSB peaks to that of polyadenylated ncRNA molecules of the *prl* class. DSB peaks map to ncRNA loci that may be situated within ORFs, near the boundaries of ORFs and intergenic regions, or most often within intergenic regions. Unconditional statistical tests revealed that this colocalization is non-random and robust (P≤5.5×10^−8^). Furthermore, we tested and rejected the hypothesis that the ncRNA loci and DSB peaks localize preferentially, but independently, to a third entity on the chromosomes.

**Conclusions/Significance:**

Meiotic DSB hotspots are directed to loci that express polyadenylated ncRNAs. This reveals an unexpected, possibly unitary mechanism for what directs meiotic recombination to hotspots. It also reveals a likely biological function for enigmatic ncRNAs. We propose specific mechanisms by which ncRNA molecules, or some aspect of RNA metabolism associated with ncRNA loci, help to position recombination protein complexes at DSB hotspots within chromosomes.

## Introduction

Over the past decade it has become clear that non-coding RNA (ncRNA) molecules have a major role in a vast array of diverse cellular processes. Small (∼20–30 nt) ncRNAs such as siRNA and miRNA can trigger the formation of euchromatin or heterochromatin, affect positively or negatively transcription, induce the deadenylation of mRNA, trigger the targeted degradation of mRNA, and regulate positively or negatively the translation of functional mRNAs (reviewed by [1], [2]). Nucleotide sequence complementarity between the small ncRNAs and target molecules serves to guide various protein complexes to the appropriate targets within mRNA (*e.g.,* for cleavage by Argonaute) [3] or within DNA of chromosomes (*e.g.,* for heterochromatinization by RITS) [4].

Another class of ncRNA molecules, the larger mRNA-like transcripts with little or no coding potential, are ubiquitous in eukaryotes ranging from fission yeast to humans [5], [6]. While the precise values are unknown and vary from organism to organism, the total number of polyadenylated ncRNAs may exceed the number of protein-coding mRNAs. With a few exceptions (*e.g.,* [7]–[10]) the function of these long ncRNAs is completely obscure, but it seems almost certain that they (like small ncRNAs) will be found to play important roles in the cell [1], [2]. We report here an unexpected, robust connection between such ncRNAs and meiotic chromosome dynamics.

**Figure 1:**
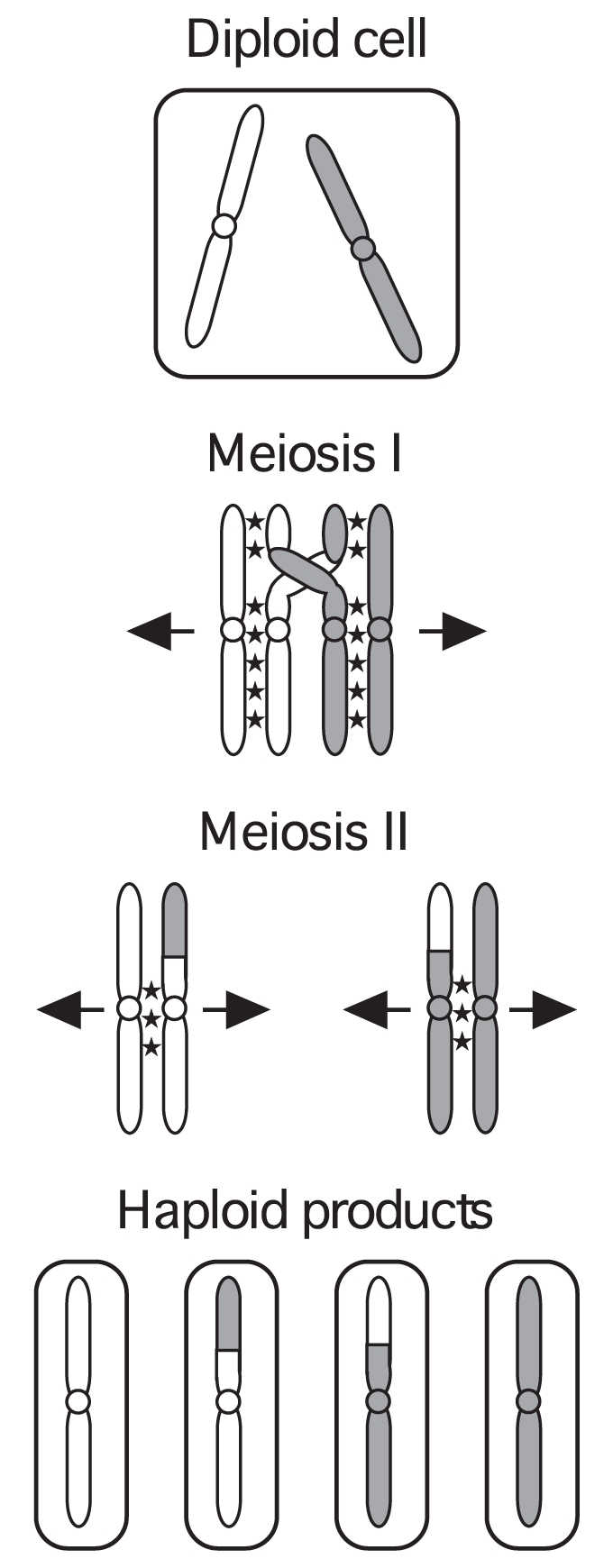
Hallmarks of meiosis. After DNA replication, homologous chromosomes (*light* and *dark*) pair and undergo a high rate of recombination. Recombination events are not distributed randomly, but cluster at hotspots. Structures created by crossover recombination (chiasmata) and sister chromatid cohesion (*stars*) facilitate the alignment and segregation of homologs in the first meiotic division. The second meiotic division is similar to mitosis, where sister chromatids segregate to opposite poles.

In meiosis, a combination of crossover recombination structures (chiasmata) and sister chromatid cohesion distal to chiasmata help to align homologous chromosome pairs and ensure their proper segregation in the first meiotic division (**Figure 1**) [11]. Consequently, meiotic recombination is not distributed randomly, but is tightly regulated to ensure that each chromosome pair receives at least one chiasma. Furthermore, recombination is positioned preferentially at hotspots along each chromosome, but current knowledge of the mechanisms for this clustering is knowledge of mechanisms for this clustering is nebulous (reviewed by [12]–[14]). The DNA binding sites for some transcription factors are hotspots [15]–[22], but there is no obvious DNA sequence preference (*i.e.,* specific consensus sequence) for hotspots across the genome [23], [24]. The relative accessibility of DNA in regions of “open” chromatin associated with transcription might facilitate the entry of meiotic recombination enzymes [16], [25]–[28], but open chromatin is insufficient to promote recombination [29] and some hotspots lack open chromatin [30], [31]. The binding of certain transcription factors [16], [22], [32], chromatin remodeling by transcription factors [25], [33], and transcription levels [19], [34], [35] either influence or are essential for local hotspot activity. Despite all of these seemingly clear connections to transcription, and paradoxically, hotspots in diverse organisms tend to cluster preferentially in non-coding regions [23], [24], [30]. It is therefore likely that additional, yet-unidentified factors help to regulate where meiotic recombination occurs.

The meiotic recombination protein Spo11 (Rec12 in fission yeast) is a conserved, topoisomerase II-like enzyme which introduces dsDNA breaks (DSBs) that initiate recombination [36]. These meiotic DSBs have been well characterized in two highly-diverged [37] organisms, budding yeast and fission yeast, and likely reflect a common mechanism for the initiation of recombination in all eukaryotes (reviewed by [14]). A recent analysis of DNA tiling microarrays revealed the genome-wide distribution of DSBs in fission yeast [23]. Most of the DSBs are clustered within 194 prominent peaks that are spaced on average about 65 kbp apart, and between the peaks there are few, if any, detectable DSBs. The DSB clusters are found in both coding regions (*i.e.,* genes) and in intergenic regions (IGRs), but they localize preferentially to large IGRs. Other than this general bias towards IGRs, no discrete features which might be responsible for DSB hotspot clustering were identified [23]. We report here that the DSB hotspots are directed preferentially to loci that express polyadenylated ncRNAs. We propose specific mechanisms by which ncRNA molecules, or features of ncRNA loci, help to regulate the positioning of meiotic recombination.

## Results

### Loci that express polyadenylated, ncRNAs are embedded within DSB peaks

**Figure 2:**
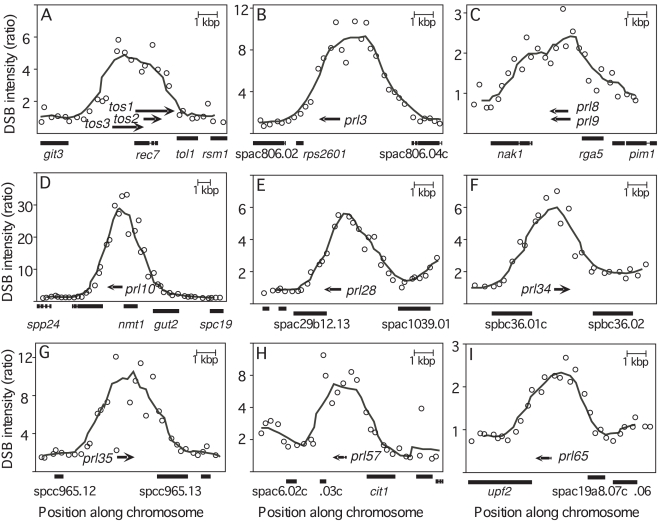
Colocalization of DSB hotspots with ncRNA. The frequencies of DSBs for each oligonucleotide tile (*circles*) and DSB peaks (*curves*) were plotted as a function of distance and are shown relative to the positions of protein-coding ORFs (*boxes*) and long ncRNAs (*tos* and *prl*, *arrowhead* indicates poly-A tail). The ncRNAs can be found within “weak” (C, I) and “prominent” (A, B, D–H) DSB peaks. Peaks and ncRNAs can map together within ORFs (A), near ORF-IGR boundaries (D, E), or within IGRs (B, C, F–I). The DSB-associated, polyadenylated ncRNAs may be spliced (H, I) or not (A–G).

Our long-term interest is to define how meiotic homologous recombination becomes localized at hotspots. We therefore examined the genomic DNA sequences surrounding meiotic DSB peaks of fission yeast, and we discovered that several of the peaks encompass DNA sequences which express ncRNA molecules. For example, a prominent DSB peak within the *rec7* gene encompasses three polyadenylated, transcript from opposite strand RNA molecules, *tos1*, *tos2*, and *tos3* (**Figure 2A**). These non-coding *tos* transcripts are induced only in meiosis [38] and are therefore present when Rec12 (Spo11) catalyses the formation of DSBs. Similarly, some prominent DSB peaks contain *prl* transcripts (polyA-bearing RNA without long open reading frames [5]) (*e.g.,* **Figure 2B**). Such non-coding *prl* transcripts are expressed in meiosis, are found within both coding regions and IGRs but localize preferentially to large IGRs, and some of them are spliced [5], [39]. In other words, the distribution and developmental regulation of some polyadenylated, ncRNA molecules seem to coincide with those of prominent DSB peaks.

### Genome-wide analyses of association between *prl* loci and DSB peaks

The fission yeast genome contains about 5,000 coding genes [40]. Among 987 cDNA clones of a random library derived from cells in mitosis and meiosis, 68 correspond to polyadenylated, ncRNAs of the *prl* class [5]. This sample is of sufficient size and complexity to be representative of the distribution and abundance of *prl* molecules expressed from the genome. We therefore compared the genome-wide distribution of DSB peaks obtained from microarray analysis [23] to that of genomic DNA sequences from which the representative *prl* molecules are transcribed [5] (**Table 1**). Since a subset of the DSB peaks were classified previously as being “prominent” (as opposed to “weak”) [23], we analyzed both the prominent peaks alone and all peaks together (prominent plus weak).

**Table 1:** Genome-wide distribution of representative *prl* loci and their proximity to DSB peaks.

				Distance from *prl* locus to DSB peaks (kbp)^2^			
Characteristics of *prl* locus expressing ncRNA^1^				Prominent peaks		All peaks	
Locus	Chr.	Position (bp)	Dir.	Left	Right	Left	Right
*prl19*	1	0051158 to 0051583	f	42.2	13.5	42.2	13.5
*prl37*	1	0071982 to 0072380	r	63.2	7.3	3.4	4.3
*prl03*	1	0237074 to 0237848	r	0.0	0.0	0.0	0.0
*prl27*	1	0299464 to 0299610	f	56.8	78.7	42.1	78.7
*prl05*	1	0434104 to 0434843	f	48.8	2.5	2.7	2.5
*prl47*	1	0491816 to 0492201	r	27.7	165.6	27.7	25.3
*prl50*	1	0977075 to 0977568	r	8.1	169.6	8.1	15.4
*prl58*	1	1393082 to 1393528	r	14.3	85.8	14.3	58.8
*prl57*	1	2327258 to 2327629	R	0.0	0.0	0.0	0.0
*prl59*	1	2362411 to 2362747	f	32.3	16.8	0.7	16.8
*prl55*	1	2416235 to 2416706	f	16.0	5.0	16.0	5.0
*prl43*	1	2432340 to 2432921	r	5.2	15.8	5.2	15.8
*prl65*	1	2472399 to 2472897	r	18.8	43.8	0.0	0.0
*prl56*	1	2885670 to 2886792	r	6.6	4.3	6.6	4.3
*prl31*	1	2975608 to 2976007	r	0.0	0.0	0.0	0.0
*prl01*	1	3003816 to 3004916	f	27.6	23.5	27.6	23.5
*prl14*	1	3190456 to 3191217	f	28.5	80.1	28.5	80.1
*prl18*	1	3699381 to 3700010	r	118.0	45.3	34.2	45.3
*prl48*	1	3942128 to 3942906	R	0.1	45.7	0.1	45.7
*prl49*	1	4008184 to 4008423	r	0.0	0.0	0.0	0.0
*prl63*	1	4008184 to 4008423	r	0.0	0.0	0.0	0.0
*prl53*	1	4008172 to 4008681	f	0.0	0.0	0.0	0.0
*prl13*	1	4259371 to 4259795	r	13.2	98.4	13.2	98.4
*prl12*	1	4439118 to 4439451	f	0.0	0.0	0.0	0.0
*prl46*	1	4651351 to 4651792	r	211.2	20.2	43.6	20.2
*prl54*	1	4820804 to 4821352	f	29.5	57.2	29.5	20.7
*prl52*	1	5023945 to 5024116	f	108.7	131.5	60.4	38.3
*prl28*	1	5441748 to 5442341	r	0.0	0.0	0.0	0.0
*prl21*	1	5454229 to 5454498	r	2.7	48.5	2.7	10.9
*prl61*	1	5546205 to 5546648	f	39.4	n.a.^3^	39.4	n.a.^3^
*prl11*	2	0028624 to 0028796	f	n.a.^3^	97.0	n.a.^3^	97.0
*prl62*	2	0227372 to 0227760	f	0.0	0.0	0.0	0.0
*prl66*	2	0547325 to 0547761	f	18.7	25.0	18.7	25.0
*prl34*	2	0835871 to 0836243	f	0.0	0.0	0.0	0.0
*prl42*	2	1032324 to 1032771	f	64.4	11.2	64.4	11.2
*prl15*	2	1329046 to 1329760	r	57.6	30.9	9.8	30.9
*prl24*	2	1497664 to 1498394	r	38.8	44.9	38.8	44.9
*prl41*	2	1652051 to 1652489	f	76.9	23.7	76.9	23.7
*prl17*	2	1777375 to 1777788	r	23.6	107.6	0.0	0.0
*plr60*	2	2005290 to 2005716	r	50.7	190.8	21.4	13.6
*prl38*	2	2186474 to 2186908	r	232.0	9.6	71.8	9.6
*prl68*	2	2204841 to 2205295	r	2.2	130.7	2.2	60.6
*prl08*	2	2485488 to 2486126	r	10.7	38.7	0.0	0.0
*prl09*	2	2485500 to 2486230	r	10.7	38.7	0.0	0.0
*prl25*	2	2567830 to 2568256	f	35.9	13.9	12.3	5.2
*prl39*	2	2804337 to 2804580	r	0.0	0.0	0.0	0.0
*prl26*	2	2827281 to 2827631	f	0.0	0.0	0.0	0.0
*prl23*	2	3120779 to 3120982	f	48.7	32.1	48.7	14.3
*prl04*	2	3295176 to 3295428	r	136.5	82.4	50.3	82.4
*prl36*	2	3313372 to 3313800	f	154.5	64.2	68.3	64.2
*prl20*	2	3934357 to 3934809	r	70.4	3.2	0.0	0.0
*prl07*	3	0041985 to 0042728	f	n.a.^3^	10.1	24.5	4.0
*prl22*	3	0084683 to 0085096	r	5.0	32.0	5.0	32.0
*prl02*	3	0394354 to 0394798	f	39.1	166.8	39.1	34.9
*prl64*	3	0827254 to 0827546	f	14.3	125.4	1.6	111.1
*prl16*	3	0882518 to 0882834	r	69.2	70.6	56.6	56.2
*prl33*	3	0953976 to 0954331	f	0.0	0.0	0.0	0.0
*prl44*	3	0955413 to 0955760	f	0.0	0.0	0.0	0.0
*prl06*	3	0968168 to 0968477	r	0.0	0.0	0.0	0.0
*prl67*	3	1432003 to 1432625	f	30.4	113.1	16.7	113.1
*prl51*	3	1514062 to 1514343	f	112.5	31.2	98.7	31.2
*prl32*	3	1702505 to 1702934	r	28.9	7.0	0.0	0.0
*prl10*	3	1837689 to 1838380	r	0.0	0.0	0.0	0.0
*prl30*	3	2009624 to 2010229	r	88.2	28.3	88.2	11.6
*prl45*	3	2299407 to 2300209	r	0.0	0.0	0.0	0.0
*prl35*	3	2310377 to 2310786	f	0.0	0.0	0.0	0.0
*prl40*	3	2391631 to 2391821	f	32.3	28.7	9.3	28.7
*prl29*	3	2419051 to 2419799	f	59.6	0.7	36.5	0.7

1 Indicate the chromosome (*Chr*.), *position*, and direction (*Dir.*) of transcription (*F*, forward; *R*, reverse) for loci from which each *prl* ncRNA is transcribed. Direction of transcription is relative to the genome sequence.

2 Distances between *prl* loci and the nearest DSB peaks (left and right) were determined as described in the methods section. Because 88% of the observed DSBs (from peak area integrals) were classified previously as falling within prominent DSB peaks [23], we analyzed the data both for prominent DSB peaks and for all DSB peaks (prominent plus weak). Ends of *prl* molecules (loci) that map within DSB peaks were assigned distance values of zero.

3 N.a., not applicable. There is no known DSB peak between the *prl* locus and the end of the chromosome, so no distance could be determined.

**Figure 3:**
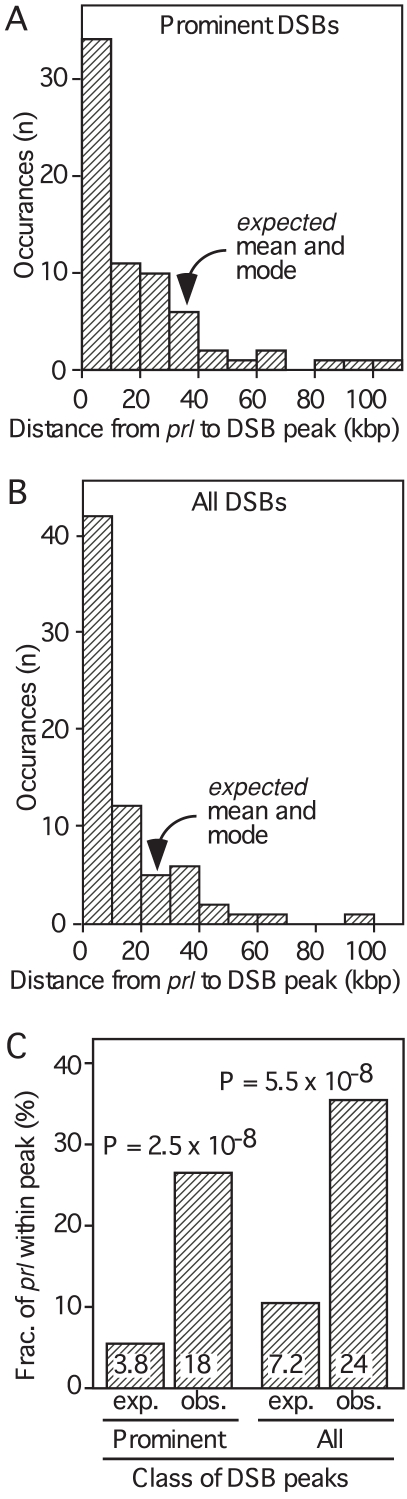
Non-random association of ncRNA with DSB hotspots. (A) Relative proximity of *prl* transcripts to prominent DSB peaks. Shown are the binned distributions of distances between each *prl* locus and the nearest DSB peak. Under the null hypothesis of no association, the mean and mode would fall in the fourth bin. (B) Relative proximity of *prl* transcripts to all (prominent and weak) DSB peaks, analyzed as in panel “A”. Under the null hypothesis, the mean and mode would fall in the third bin. (C) Non-random colocalization of DSB peaks with ncRNA of the *prl* class. Plot shows the expected (*exp*) and observed (*obs*) values for the localization of *prl* molecules entirely within DSB peaks.

The average distance between prominent DSB peaks is 65 kbp and the average distance between the representative *prl* loci is 185 kbp. One might expect these *prl* loci to map, on average, about 33 kbp away from DSB peaks. However, the distribution of distances between *prl* loci and their neighboring DSB peaks is skewed markedly towards a much shorter distance (**Figure 3A–B**). Furthermore, there is an unexpectedly high frequency of perfect colocalization (**Figure 2**, **Table 1**): Of the 68 ncRNA molecules analyzed, 18 (26.5%) map entirely within prominent DSB peaks. Six additional ncRNAs (8.8%) map entirely within weak DSB peaks. Fourteen more (20.5%) fall on or near the flanks of DSB peaks (within 5 kbp). In toto, about 56% of the representative ncRNA loci are associated with DSB peaks by these criteria. To analyze these data further, we calculated the unconditional genome-wide probabilities of perfect colocalization, which are the most conservative and rigorous criteria possible.

The 194 prominent DSB peaks encompass about 700 kbp (5.6%) of the genome sequence and those peaks are separated by regions essentially devoid of DSBs [23]. One can view the ncRNA molecules as having a binomial distribution with respect to landing successfully (yes or no) within a DSB peak. We therefore analyzed the data using the exact binomial test of a single proportion. The probability of success under the null hypothesis of random placement anywhere within the genome would be 5.6% per molecule. This yields an expected value of 3.8 out of 68 *prl* molecules falling within a prominent DSB peak, and 96.4% confidence that the number of molecules within a prominent peak should be seven or less. The probability of observing 18 or more *prl* molecules out of 68 landing within a prominent DSB peak by chance is low (P = 2.5×10^−8^) (**Figure 3C**). We conclude that there is a positive, non-random association between loci which express the *prl* class of ncRNA molecules and the positions of prominent DSB clusters that initiate meiotic recombination.

The preceding analyses considered the 194 prominent DSB peaks that collectively contain most of the meiotic DSBs catalyzed by Rec12 (Spo11). However, there are an additional 159 “weak” DSB peaks that encompass less than 5% of the genome and whose aggregate area integrals contribute 12% of the total DSBs measured across the genome [23]. Within these weak peaks we found six additional ncRNA transcripts of the *prl* data set (*e.g.,* **Figure 2C, I**). Therefore 35.3% (24 of 68) of the *prl* molecules fall entirely within a DSB peak (weak or prominent). The complete set of all detectable DSB peaks (weak and prominent) map to about 10.6% of the genome sequence [23]. Under a null-hypothesis success probability of 10.6% per molecule, one would expect 7.2 of the 68 *prl* molecules to fall within a peak by chance, and have 97.4% confidence that the number falling within a peak would be 12 or less. The probability of observing 24 or more occurrences out of 68 is low (P = 5.5×10^−8^) (**Figure 3C**). We conclude that there is a positive, non-random association between *prl* loci and DSB peaks for the entire contingent of all known DSB peaks (prominent and weak) across the genome.

### Test of an alternative hypothesis: Independent localization to IGRs?

About 81% of the prominent DSB peaks, 61% of the weak DSB peaks, and 87% of the representative *prl* loci are within IGRs [5], [23]. This raises the possibility that the apparent non-random associations noted above arise simply from *prl* loci and DSB peaks localizing preferentially, but independently, to IGRs. To explore this possibility further, we restricted the analysis to the 40% of the fission yeast genome comprised of IGRs, and we computed the corresponding conditional probabilities under the binomial distribution and null hypothesis of no association. DSB peaks (prominent and weak) cover 22% of DNA within IGRs, prominent peaks cover 14% of DNA within IGRs, and 59 *prl* loci are specific to IGRs. Twenty four of the 59 IGR-specific *prl* loci (41%) occur within a DSB peak (P = 9.6×10^−4^), 18 of which (31%) occur within a prominent peak (P = 8.6×10^−4^). We conclude that the intimate association between DSB peaks and *prl* loci does not arise from independently preferential localization of each to an IGR. Rather, the DSB peaks colocalize specifically with ncRNA loci that may be situated within ORFs, near ORF-IGR boundaries, or most often within IGRs (*e.g.,* **Figure 2**).

## Discussion

It is implausible, statistically, that the association between DSB peaks and ncRNAs of the *prl* class is coincidental (P≤5.5×10^−8^, **Figure 3C**). The strength of this association was documented using the most strict criteria possible (perfect colocalization) and it takes into account all of those instances where DSB peaks and representative ncRNA loci do not colocalize precisely. It applies whether one examines only the prominent DSB peaks or all known DSB peaks. Moreover, we tested and rejected the hypothesis that *prl* loci and DSB peaks localize preferentially, but independently, to a third entity on the chromosomes. Together these findings support an unambiguous, overall conclusion—meiotic DSB hotspots of fission yeast are directed preferentially to loci that express long, polyadenylated ncRNA molecules of the *prl* class.

### DSB hotspots localize to ncRNA loci far more intimately than to any other known factors

The genome-wide, preferential localization of DSB peaks to representative ncRNA loci is unconditional and robust (P≤5.5×10^−8^, **Figure 3C**). For comparison, three other factors are reported to correlate positively with the genome-wide distribution of DSB hotpots, at least under conditional parameters [23], [24]. In both fission yeast and budding yeast, DSB peaks are associated with a slightly elevated G:C content (P>0.05 to P≤0.0001, depending upon window size and the other conditional parameters applied). In both yeasts there is also an association of DSB peaks with those IGRs located 5′ of protein-coding genes that are divergently transcribed (P = 0.001 to P≤0.0001, from conditional assessment of IGRs alone). This correlation breaks down (P>0.05) in fission yeast when the size of the IGR is controlled for; such IGR size controls were not reported for budding yeast. And in fission yeast, the presumptive promoter regions of genes with DSB peaks are enriched for gene ontology (GO) terms associated with “interaction between organisms” or “transcription factor activity” (from conditional assessment of presumtive promoter regions alone, no P values reported). Among these various factors, the presence of loci expressing the polyadenylated *prl* class of ncRNA is by far the single best predictor of where meiotic DSB peaks localize in the genome (P≤5.5×10^−8^, **Figure 3C**).

Transcripts of the *prl* class are expressed in meiosis (many exclusively in meiosis) [5], [38], [39], so they are present at the developmental stage in which Rec12 (Spo11) catalyzes the formation of DSBs. We therefore propose that ncRNA molecules or loci of the *prl* class help to activate DSB hotspots. This process may be conserved, because in mice one ncRNA has been shown to map to a well-defined meiotic recombination hotspot [41] and at least one other has been implicated to do so [42].

### Minimum and maximum estimates of potency

The vast majority of fission yeast ncRNAs remain undiscovered [5], so the presence and frequency of ncRNA-free DSB peaks is uninformative scientifically. We note, however, that there are enough predicted ncRNA loci [5] to populate each of the known DSB peaks.

About 35% of the representative *prl* loci are embedded entirely within DSB peaks (**Figures 2** **–** **3**, **Table 1**), so if one excludes any possible function at distance the remaining 65% of *prl* loci would not be sufficient to promote recombination. However, two factors suggest that the fraction of potentially recombinogenic *prl* loci may be much greater than 35%. First, the distribution of DSB peaks in fission yeast was determined using *rad50S* strains [23], and in *rad50S* strains of budding yeast about half of all meiotic DSB peaks escape detection [43], [44]. If this applies to fission yeast, then there would be about twice as many DSB peaks as reported. In that case, somewhere between at least 35% (the current observed value) and approximately 70% (an extrapolated value) of the representative *prl* loci would fall entirely within DSB peaks. Second, if one allows for possible function at even a very short distance (*e.g.,* ≤5 kbp), then the fraction of *prl* loci associated with DSB peaks also increases dramatically (by 58%, **Table 1**). Such possible function at distance is indicated clearly by the data (means and modes, **Figure 3A–B**). For these reasons, the theoretical maximum value for functional association may approach unity.

### Hypothetical mechanisms: Chromatin structure or guide RNA

How might long, polyadenylated ncRNA molecules or loci that express ncRNA molecules help to position the initiation of meiotic recombination catalyzed by Rec12 (Spo11)? We propose two, not mutually exclusive, hypotheses on mechanism.

#### A chromatin docking-site hypothesis for hotspot meiotic recombination at ncRNA loci

In fission yeast meiotically induced chromatin remodeling occurs at DSB hotspots [45], [46] and where tested is seemingly required for hotspot activity [25]. Therefore one possibility is that *prl*-dependent chromatin remodeling, due either to some aspect of transcription at *prl* loci or mediated by the ncRNA molecules themselves, creates a preferential site for the nucleation of recombination protein complexes. This hypothesis fits comfortably within the prevailing orthodoxy, which posits that the relative accessibility of DNA in regions of “open” chromatin has a role in hotspot activity (reviewed by [12], [14], [47], [48]).

#### A guide RNA hypothesis for hotspot meiotic recombination

Small ncRNAs (*e.g.,* siRNA) base pair with their targets (RNA or DNA) and thereby deliver protein complexes to those targets [3], [4]. Similarly, ncRNA-DNA hybrids (R-loops) formed by long ncRNAs are implicated to direct the positioning of class switch recombination during B cell maturation [10], [49], [50]. We propose that an analogous mechanism operates to direct the machinery of meiotic recombination to ncRNA loci within chromosomes. This may involve a homology search of DNA by protein-RNA complexes (*e.g.,* as is implicated for the siRNA-containing RITS complex). Alternatively, it may involve the recognition of R-loop structures by protein complexes (*e.g.,* as is implicated for class switch recombination). In either case, the base pairing between ncRNA molecules and homologous chromosomal DNA would guide recombination to hotspots.

We emphasize that our proposals are not mutually exclusive with other hypothetical mechanisms proposed previously (reviewed by [12]–[14]) and the models even overlap to some extent. For example, R-loops would impart changes in local nucleosome organization and alter the sensitivity of DNA within chromatin to nucleases, which is a hallmark of most recombination hotspots and is invoked as a feature of most current models.

### Implications and context

Our findings have two main implications. First, they reveal an unexpected, potentially unitary mechanism for what directs meiotic recombination to hotspots (expressed ncRNA loci). Second, they reveal a likely biological function for many of the enigmatic, polyadenylated ncRNA molecules that are so abundant in eukaryotes.

One of our two alternative hypotheses is that the polyadenylated ncRNA molecules help to position meiotic recombination. For context, this hypothesis is consistent with, and might explain mechanistically, some of the many connections between transcription and recombination documented previously (see Introduction). A few additional examples are provide here. Hotspot-activating RNA molecules would in principle confer differential DNA strand identity at hotspots, and hence could provide an underlying basis for the seemingly asymmetrical nature of all DSBs (directionality) [51] and for the preferential transfer of one DNA strand into heteroduplex DNA (strand identity) [52]. Similarly, our hypothesis may explain why the known RNA metabolism protein Ski8 (Rec14 of fission yeast) is an essential component of the Spo11 (Rec12) meiotic recombination protein complex in several organisms ([53]–[55] and our unpublished observations).

## Materials and Methods

### Relative proximity of DSB peaks to *prl* loci

Sixty-eight polyadenylated, ncRNA molecules of the *prl* class were identified among 987 random cDNA clones [5]. ORF [40], *tos* [38], and *prl* [5] locations were obtained from the fission yeast genome database at the Sanger institute [40]. Raw data on the genome-wide distribution of meiotic DSBs from dataset S1 [23] were analyzed with permission (Creative Commons Attribution License). Raw experimental data were averaged and divided by those of a negative, mitotic control to yield DSB intensity ratio, and peak curves for display were drawn using a sliding, 5-point average. Where available, we used the *prl* locations annotated in the Agilent microarray spreadsheet (Agilent Technologies). Seventeen *prl* loci were not annotated in that spreadsheet (*prl7*, *prl9*, *prl11*, *prl15*, *prl17*, *prl18*, *prl22*, *prl25*, *prl27*, *prl29*, *prl30*, *prl49*, *prl56*, *prl61*, *prl63*, *prl64*, *plr67*). These were added manually. One locus (*prl52*) was annotated twice (on chromosomes I and III). The position for chromosome I was used, as it is congruent with the location listed in the genome database. The distances between each *prl* locus and its two neighboring DSB peaks (left and right) were determined and tabulated in Excel 2005 (Microsoft Corporation, Redmond, WA). Ends of *prl* molecules (loci) that map within DSB peaks were assigned distance values of zero. Because 88% of the observed DSBs (from area integrals) were classified previously as falling within prominent DSB peaks [23], we analyzed the data both for prominent DSB peaks and for all DSB peaks (prominent plus weak) (**Table 1**).

### Statistical measures

Each locus expressing an ncRNA molecule can map either within or outside of a DSB peak. Thus, under random genome-wide placement with constant per-locus “success” probability of mapping within a peak (the null hypothesis), the set of ncRNA loci should approximate the binomial distribution with respect to DSB peaks. We therefore modeled the data using the Binomial distribution. Binomial proportion parameters (success probabilities per locus) were estimated as equal to the fraction of the genome encompassed by “all” and “prominent” DSB peaks. Conditional binomial proportion parameters (success probabilities per locus, given that the locus is within an IGR) were similarly set equal to the fraction of IGRs encompassed by “all” and “prominent” DSB peaks. For every parameter estimate, the *P* value for the observed number of mappings to DSB peaks was calculated using the exact binomial test of a single proportion. Exact binomial one-sided upper confidence limits were also calculated for the expected number of successes under the null hypothesis. Calculations were performed using the “BINOMDIST” function in a Microsoft Excel spreadsheet (Microsoft Corporation, Redmond, WA). The binomial-proportion estimates, sample sizes, observed success rates, null-hypothesis confidence limits, and calculated *P* values for each test scenario are given in the main text.
